# Changes in DNA methylation and transgenerational mobilization of a transposable element (*mPing*) by the Topoisomerase II inhibitor, Etoposide, in rice

**DOI:** 10.1186/1471-2229-12-48

**Published:** 2012-04-09

**Authors:** Xuejiao Yang, Yingjie Yu, Lily Jiang, Xiuyun Lin, Chunyu Zhang, Xiufang Ou, Kenji Osabe, Bao Liu

**Affiliations:** 1 Key Laboratory of Molecular Epigenetics of MOE, and Institute of Genetics & Cytology, Northeast Normal University, Changchun, 130024, China; 2 Jilin Academy of Agricultural Sciences, Changchun, 130033, China; 3 CSIRO Plant Industry, Canberra, ACT, 2601, Australia

## Abstract

**Background:**

Etoposide (epipodophyllotoxin) is a chemical commonly used as an anti-cancer drug which inhibits DNA synthesis by blocking topoisomerase II activity. Previous studies in animal cells have demonstrated that etoposide constitutes a genotoxic stress which may induce genomic instability including mobilization of normally quiescent transposable elements (TEs). However, it remained unknown whether similar genetically mutagenic effects could be imposed by etoposide in plant cells. Also, no information is available with regard to whether the drug may cause a perturbation of epigenetic stability in any organism.

**Results:**

To investigate whether etoposide could generate genetic and/or epigenetic instability in plant cells, we applied etoposide to germinating seeds of six cultivated rice (*Oryza sativa* L.) genotypes including both subspecies, *japonica* and *indica*. Based on the methylation-sensitive gel-blotting results, epigenetic changes in DNA methylation of three TEs (*Tos17*, *Osr23* and *Osr36*) and two protein-encoding genes (*Homeobox* and *CDPK-related* genes) were detected in the etoposide-treated plants (S0 generation) in four of the six studied *japonica* cultivars, Nipponbare, RZ1, RZ2, and RZ35, but not in the rest *japonica* cultivar (Matsumae) and the *indica* cultivar (93-11). DNA methylation changes in the etoposide-treated S0 rice plants were validated by bisulfite sequencing at both of two analyzed loci (*Tos17* and *Osr36*). Transpositional activity was tested for eight TEs endogenous to the rice genome in both the S0 plants and their selfed progenies (S1 and S2) of one of the cultivars, RZ1, which manifested heritable phenotypic variations. Results indicated that no transposition occurred in the etoposide-treated S0 plants for any of the TEs. Nonetheless, a MITE transposon, *mPing*, showed rampant mobilization in the S1 and S2 progenies descended from the drug-treated S0 plants.

**Conclusions:**

Our results demonstrate that etoposide imposes a similar genotoxic stress on plant cells as it does on animal and human cells, which may induce transgenerational genomic instability by instigating transpositional activation of otherwise dormant TEs. In addition, we show for the first time that etoposide may induce epigenetic instability in the form of altered DNA methylation patterns in eukaryotes. However, penetrance of the genotoxic effects of etoposide on plant cells, as being reflected as genetic and epigenetic instability, appears to be in a strictly genotype- and/or generation-dependent manner.

## Background

Etoposide (epipodophyllotoxin) is a chemical which has been used widely as an anti-cancer drug as it inhibits DNA synthesis by forming a complex with topoisomerase II [1]. During DNA replication, topoisomerase II can break, unwind and repair both strands of the double-stranded DNA that is supercoiled during the unwinding process, hence releasing the tension built up on the supercoiled DNA and repairing topoisomerase II-associated double-stranded DNA breaks (DSBs) [1]. Accordingly, inhibition of topoisomerase II activity accumulates breaks in DNA, prevents entry into the mitotic phase of cell division, and leads to cell death. Etoposide acts primarily in the G2 and S phases of the cell cycle [2].

Apart from directly generating DSBs, previous studies in animal cells have also shown that etoposide constitutes a genotoxic stress which may induce genomic instability indirectly by instigating mobility of otherwise quiescent transposable elements (TEs), and hence generating insertional mutagenesis [3]. Most plant genomes harbor a large proportion of TEs and some of which are known as inducible to become transcriptionally active and even transpositionally mobile under stress conditions [4]. Therefore, it is interesting to explore whether etoposide treatments may produce similar effects on the activity of TEs in plant genomes, as no information has been available in this aspect.

It becomes increasingly clear that genetic information encoded in the primary DNA sequence is not the only determinant of heritable phenotypes. Epigenetic modifications, collectively known as epigenome, also participate in orchestrating gene expression as well as in maintaining genomic stability [5]. Among the epigenetic modifications, cytosine DNA methylation is the best studied, and plays essential roles as an evolutionarily conserved genome defense device as well as a master regulator of genome-wide temporal and spatial gene expression [6,7]. More importantly, DNA methylation is sensitive and responsive to environmental cues including genotoxic stress and may generate new and heritable epialleles coping with the particular environmental condition accordingly [8-11]. For instance, it was found in plant cells that various environmental conditions can induce DNA methylation alterations, and those progenies inheriting the altered methylation patterns exhibited enhanced tolerance to the specific stress their progenitors experienced [12-14]. Given the above, it is of apparent interest to test if the genotoxic stress imposed by etoposide may induce epigenetic instability in the form of DNA methylation changes. Surprisingly, however, to our knowledge, there is no report on the epigenetic effects of etoposide treatments in any organism.

The aims of this investigation were to (1) test whether the topoisomerase II inhibitor, etoposide may induce epigenetic instability in the form of DNA methylation changes; (2) explore whether etoposide may also instigate mobilization of transposable elements (TEs) in plant cells. We addressed these two questions in six cultivated genotypes of rice (*Oryza sativa* L.) representing both subspecies, *japonica* and *indica*.

## Results

### Etoposide-induced epigenetic changes in the form of DNA methylation were manifested in somatic cells of the immediately treated S0 rice plants

For all six studied rice genotypes, the seedling plants treated with etoposide at both concentrations (10 and 20 mg/L, respectively) appeared normal apart from a slight retardation in the overall statue (data not shown), and no difference was observed between the two concentrations. Moreover, after a two-month period of recovering, no discernible difference was observable between the treated and the control plants. This suggests that physiological toxicity to the rice seedlings by the chemical was mild and temporary at the studied concentrations. Nonetheless, it is known from previous studies that some abiotic and genotoxic stresses may impose longer-term or even transgenerational genomic and/or epigenomic effects without expressing immediate phenotypic or physiological effects.

To test whether the etoposide-imposed genotoxic stress may induce changes in cytosine DNA methylation in somatic cells of the immediately treated rice plants (designated as S0) that showed no evidence of phenotypic abnormality, we performed methylation-sensitive Southern blotting with a set of 15 pre-selected probes representing both transposable elements (TEs) and known-function protein-encoding genes, the intrinsic methylation states of which are known to be variable in the wild-type rice genome, i.e., being heavily or moderately methylated or unmethylated, respectively [15-17]. DNA of pooled plants (10–15 individuals) for each genotype was analyzed. Thus, if methylation repatterning would occur due to etoposide treatments, then, both hypo- and hypermethylation could be expected in the gel-blotting patterns by one or more types of these probes on genomic DNAs restricted by the methylation-sensitive endonucleases.

**Figure 1:**
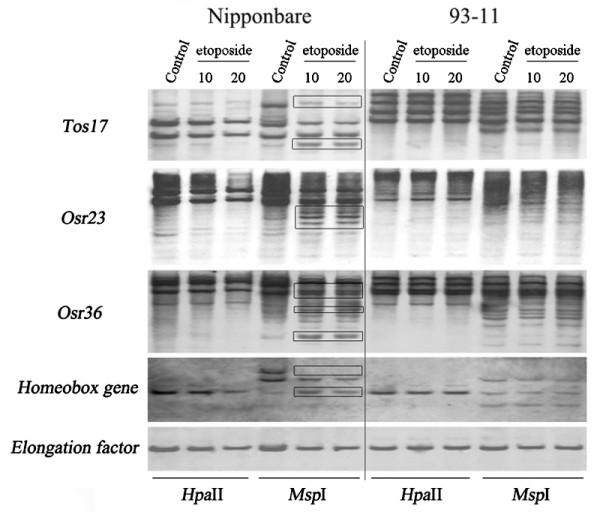
**Changes in DNA methylation in the S0 generation of etoposide- treated plants of genotypes Nipponbare and 93-11 relative to their respective wild-type control plants.** Each genotype was treated with two concentrations of etoposide, 10 mg/L and 20 mg/L, respectively. “Control” represents plants treated with ddH_2_O. DNA methylation changes occurred only at the CHG context of the 5’-CCGG sites (in *Msp*I digestion, denoted by rectangles), and the changes occurred only in Nipponbare. Note that the two etoposide concentrations showed the same changes. The *Elongation factor* gene is intrinsically unmethylated in the rice genome and thus showed a single monomorphic band among all treated plants in both *Hpa*II and *Msp*I digestions of each genotype. This monomorphic hybridizing pattern of *Elongation factor* also validated complete digestion by the methylation-sensitive isoschizomers in all DNA samples.

**Figure 2:**
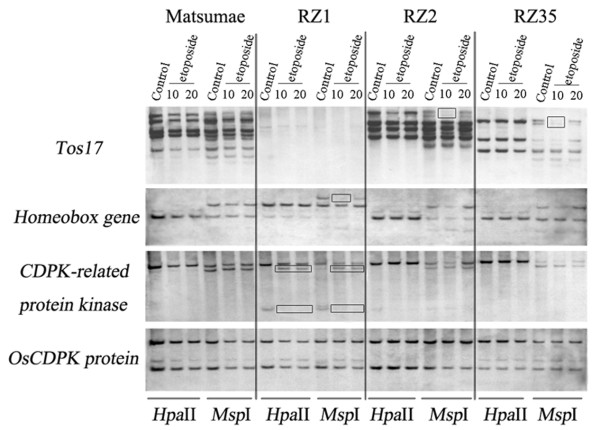
**Changes in DNA methylation in the S0 generation of etoposide- treated plants of genotypes Matsumae, RZ1, RZ2 and RZ35, relative to their respective control plants.** Each genotype was treated by two concentrations of etoposide, 10 mg/L and 20 mg/L, respectively. “Control” represents plants treated with ddH_2_O. Notably, DNA methylation changes occurred in all three recombinant inbred lines (RZ1, RZ2 and RZ35) but not in their rice parental line Matsumae. Also, the changes occurred in either or both of the enzyme digests, though more abundantly in *Msp*I-digest, and the changes occurred either in the 10 mg/L concentration or in both concentrations. The *OsCDPK* protein-encoding gene is intrinsically unmethylated and thus showed a monomorphic banding pattern among all treated plants in both *Hpa*II and *Msp*I digestions across the genotypes, indicating complete digestion in all samples.

Indeed, the methylation-sensitive Southern blotting patterns indicated that of the six rice genotypes, four showed DNA methylation changes in the etoposide-treated plants vs. their respective controls in at least one of the 15 tested probes (Table 1). Specifically, the following results were obtained: (1) For the two laboratory standard rice genotypes representing respectively the *japonica* and *indica* subspecies, Nipponbare and 93-11, only the former showed methylation changes in four (three TEs and one gene) of the 15 probes, and most of the changes belonged to decrease in methylation, i.e., hypomethylation at the CHG sites of the 5’-CCGG tetranucleotide(s) within or immediately flanking the probe sequences (i.e., changes only occurred in *Msp*I digest) (Table 1; Figure 1). (2) For the three RILs (RZ1, RZ2 and RZ35) and their rice parental line (Matsumae), all three RILs showed methylation changes in one to three (two TEs and two genes) of the 15 probes, while Matsumae did not show any change (Table 1; Figure 2). An interesting observation common to the DNA methylation changes in these rice genotypes was that little difference was detected between the two concentrations (10 and 20 mg/L) of the etoposide treatment (Table 1; Figures 1 and 2).

**Table 1:** DNA methylation alterations of TEs and protein-encoding genes in the S0 generation of etoposide-treated plants relative to the controls, detected by methylation-sensitive gel-blotting in various rice genotypes

**Probe**	**GenBank Accession No.^a^**	**DNA methylation changes in Etoposide-treated rice plants (S0)**											
		**Nipponbare**		**93-11**		**Matsumae**		**RZ1**		**RZ2**		**RZ35**	
		**10 mg/L** **(H/M)^b^**	**20 mg/L** **(H/M)**	**10 mg/L** **(H/M)**	**20 mg/L** **(H/M)**	**10 mg/L** **(H/M)**	**20 mg/L** **(H/M)**	**10 mg/L** **(H/M)**	**20 mg/L** **(H/M)**	**10 mg/L** **(H/M)**	**20 mg/L** **(H/M)**	**10 mg/L** **(H/M)**	**20 mg/L** **(H/M)**
**Transposable elements (TEs)**													
*Tos17*	AC087545	n/-	n/-	n/n	n/n	n/n	n/n	n/n	n/n	n/-	n/n	n/-	n/n
*Osr2*	AL442110	n/n	n/n	n/n	n/n	n/n	n/n	n/n	n/n	n/n	n/n	n/n	n/n
*Osr23*	AP002843	n/+	n/+	n/n	n/n	n/n	n/n	n/n	n/n	n/n	n/n	n/n	n/n
*Osr35*	AC068924	n/n	n/n	n/n	n/n	n/n	n/n	n/n	n/n	n/n	n/n	n/n	n/n
*Osr36*	AP001551	n/-	n/-	n/n	n/n	n/n	n/n	n/+	n/+	n/n	n/n	n/n	n/n
*Ping-specific*	AB087616	n/n	n/n	n/n	n/n	n/n	n/n	n/n	n/n	n/n	n/n	n/n	n/n
*Pong-specific*	BK000586	n/n	n/n	n/n	n/n	n/n	n/n	n/n	n/n	n/n	n/n	n/n	n/n
*mPing*	AP005628	n/n	n/n	n/n	n/n	n/n	n/n	n/n	n/n	n/n	n/n	n/n	n/n
**Protein-encoding genes**													
*Homeobox gene*	AB007627	n/-	n/-	n/n	n/n	n/n	n/n	n/-	n/n	n/n	n/n	n/n	n/n
*Binding*	X88798	n/n	n/n	n/n	n/n	n/n	n/n	n/n	n/n	n/n	n/n	n/n	n/n
*CAL-2*	AK069341	n/n	n/n	n/n	n/n	n/n	n/n	n/n	n/n	n/n	n/n	n/n	n/n
*CAL-11*	X81393	n/n	n/n	n/n	n/n	n/n	n/n	n/n	n/n	n/n	n/n	n/n	n/n
*Elongation factor*	D12821	n/n	n/n	n/n	n/n	n/n	n/n	n/n	n/n	n/n	n/n	n/n	n/n
*OsCDPK protein*	AY144497	n/n	n/n	n/n	n/n	n/n	n/n	n/n	n/n	n/n	n/n	n/n	n/n
*CDPK-related protein kinase*	AP004380	n/n	n/n	n/n	n/n	n/n	n/n	+/+	+/+	n/n	n/n	n/n	n/n
Variation frequency(%)^c^		26.67	26.67	0	0	0	0	20	13.33	6.67	0	6.67	0

^a^ Determined by BlastN at NCBI.

^b^ Digestions by *Hpa*II (H) and *Msp*I (M) were separated by a slash “/”; n: no change in cytosine methylation; +: increase in cytosine methylation; -: decrease in cytosine methylation; *Hpa*II is related to the methylation alteration at CG sites, *Msp*I is related to the methylation alteration at CHG sites.

^c^ Defined as the number of probes showing alterations/the total number of analyzed probes.

### Further analysis and validation of the etoposide-induced DNA methylation changes in the S0 rice plants by bisulfite sequencing

**Figure 3:**
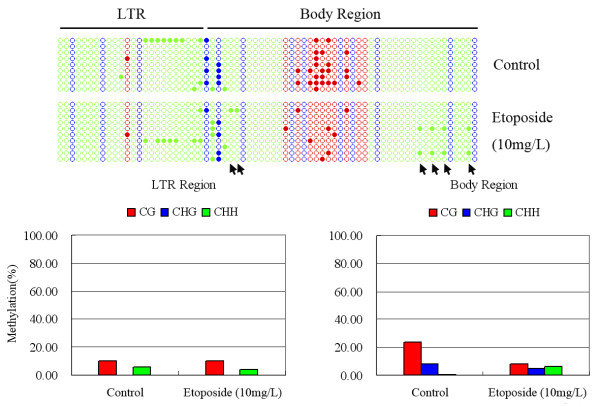
**Cytosine methylation changes in a fragment of *Tos17 *(encompassing portions of the 5'-LTR- and body-region) in the S0 generation of the etoposide-treated plants of Nipponbare, determined by bisulfite sequencing.** Nine and 10 clones were arbitrarily sequenced for the control and the 10 mg/L etoposide-treated plants, respectively. All three types of cytosines, CG (red circles), CHG (blue circles) and CHH (green circles) were shown in the map. Filled and empty circles denote methylated and unmethylated cytosines, respectively. The red, blue and green columns in the histograms refer to the collective methylation levels (in percentage) respectively of CG, CHG and CHH, of the LTR- and body-regions. The nucleotide sequence of this analyzed fragment was presented in Additional file 1. *De novo* methylation at six CHH sites as a result of the etoposide-treatment were arrowed.

**Figure 4:**
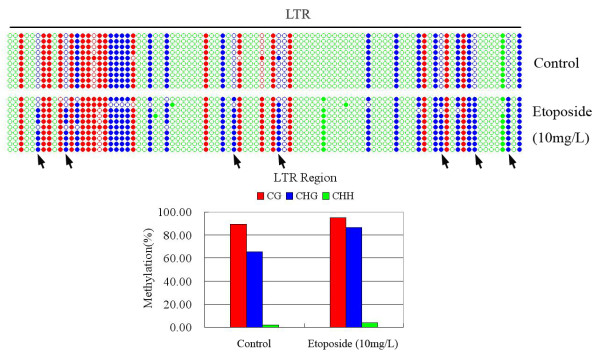
**Cytosine methylation changes in a fragment of *Osr36 *(portion of the 5'-LTR-region) in the S0 generation of the etoposide-treated plants of Nipponbare, determined by bisulfite sequencing**. Ten clones were arbitrarily sequenced for the control and the 10 mg/L etoposide-treated plants, respectively. All three types of cytosines, CG (red circles), CHG (blue circles) and CHH (green circles) were shown in the map. Filled and empty circles denote methylated and unmethylated cytosines, respectively. The red, blue and green columns in the histograms refer to the collective methylation levels (in percentage) respectively of CG, CHG and CHH, of the LTR-region. The seven CHG sites that showed dramatic hypermethylation were arrowed. The nucleotide sequence of this analyzed fragment was presented in Additional file 1.

The methylation-sensitive Southern blotting analysis was capable of only revealing methylation changes at the cytosines that were within or immediately adjacent to the enzyme recognition/restriction site(s). In this case, methylation changes only at the two cytosines (inner and outer) of the 5’-CCGG tetranucleotide(s) were detectable, as which were recognition/restriction sites of the pair of isoschizomers, *Hpa*II/*Msp*I. To further explore whether changes in methylation also occurred at other cytosines, as well as to validate the blotting-detected methylation changes, by an independent approach, we performed bisulfite sequencing for two segments representing the two TEs (*Tos17* and *Osr36*) that showed clear methylation changes in the etoposide-treated S0 plants of Nipponbare (Figure 1). For *Tos17*, we designed bisulfite sequencing primers that encompassed portion of the 5’-LTR and portion of the internal body-region of the retroelement. The bisulfite sequencing results showed that whereas the LTR portion was intrinsically hypomethylated at all three types of cytosine sites, CG, CHG and CHH, the body-region was moderately methylated at the CG and CHG sites but virtually unmethylated at the CHH sites in the wild-type rice genome; the LTR region did not show discernibly gain of methylation subsequent to the etoposide treatment (10 mg/L), but substantial reduction of CG methylation and minor reduction of CHG methylation occurred in the body-region of the retroelement due to the etoposide treatment, which was accompanied by some *de novo* hypermethylation at a few CHH sites (Figure 3). The bisulfite sequencing results for this segment were in broad agreement with the methylation-sensitive gel-blotting results (Figure 1). For *Osr36*, we designed bisulfite sequencing primers within the 5’-LTR region of this retroelement, because this region was known to be heavily methylated according to our previous gel-blotting results in other rice genotypes [18]. The bisulfite sequencing results indeed showed that in the wild-type control plants of Nipponbare, the CG and CHG sites were heavily methylated in the segment whereas the CHH sites were nearly completely (except for one or two positions) devoid of methylation. Only moderate methylation changes were detected in the etoposide-treated plants at the CG sites, but substantial changes were detected at the CHG sites (Figure 4). Surprisingly, in contrast with the changes in this TE detected in the gel-blotting which showed hypomethylation (Figure 1), the collective changes (all cytosines of a given type being considered together) revealed by bisulfite sequencing at both CG and CHG sites represented hypermethylation (Figure 4). Given that the gel-blotting was only capable of detecting methylation changes at the 5-CCGG sites, whereas the bisulfite sequencing results were the collective of all cytosines within the sequenced region, the seemingly discrepant results can be readily reconciled (Figure 4).

### Transposon *mPing* was totally quiescent in the etoposide-treated S0 plants, but showed rampant transgenerational mobilization in their S1 and S2 progenies in one of the genotypes, RZ1

**Figure 5:**
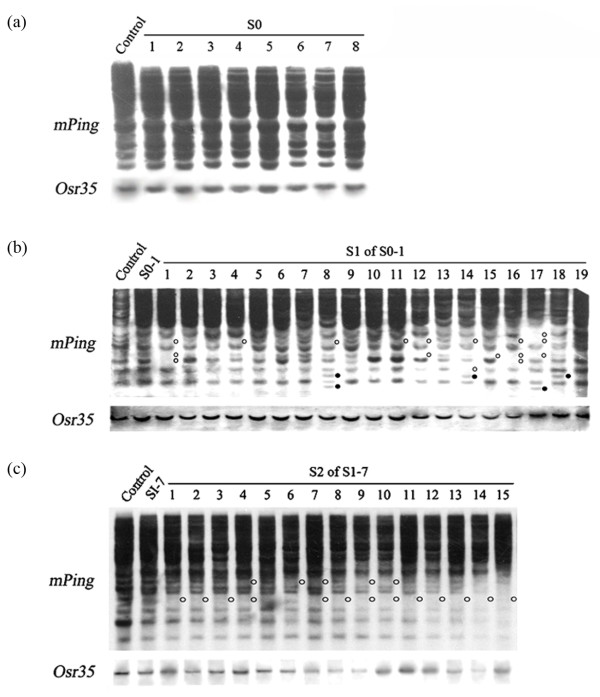
**Stability or mobilization of *mPing *in different selfed generations of the etoposide-treated plants of RZ1, revealed by DNA gel-blotting. (a)** Complete stability of *mPing* was observed in eight randomly chosen etoposide-treated S0 plants. Also, no evidence for mobility was detected for a set of low-copy LTR retrotransposons (e.g., *Osr35*) in these S0 plants. **(b)** Mobilization of *mPing* was detected in at least 10 of the 19 randomly chosen individual plants of the S1 generation derived from a single S0 plant (S0-1). The empty and solid circles denote excision and reinsertion events, respectively. No evidence for mobility was detected for a set of low-copy LTR retrotransposons (e.g., *Osr35*) in any of these S1 plants. **(c)** Additional transposition of *mPing* was detected in 14 of the 15 S2 plants derived from a single S1 individual (S1-7) in which no transposition of this TE was detected. Only excisions were detected in these plants (marked by empty circles). Again, no evidence for mobility was detected for a set of low-copy LTR retrotransposons (e.g., *Osr35*) in any of these S2 plants. The DNAs in these blots were digested by *Xba*I.

Whole genome sequencing has revealed that a substantial portion of plant genomes is comprised of transposable elements (TEs) and their derivatives, and a small fraction of the TEs still possesses the ability to become transpositionally active (mobile) under specific stress conditions, and which are often accompanied by epigenetic remodeling [19-21]. The most labile TEs to become mobile in the rice genome are the MITE *mPing*, its autonomous TPase donors *Ping* and *Pong*, and a set of low-copy, *copia*-like, LTR retrotransposons including *Tos17*, *Osr23*, *Osr35*, *Osr36* and *Osr42 *[21,22]. Given that the etoposide-treated S0 rice plants showed changes in DNA methylation, it was interesting to test whether some of these potentially mobile TEs might become active in these plants. We therefore conducted gel-blotting for these eight TEs (*mPing*, *Ping*, *Pong*, *Tos17*, *Osr23*, *Osr35*, *Osr36* and *Osr42*). We used randomly chosen individual plants of three consecutive generations, S0 (n = 8), S1 (n = 19) and S2 (n = 15), of genotype RZ1 to study this issue because these plants showed heritable phenotypic variations (detailed below). We found that all these TEs showed a monomorphic pattern among the eight etoposide-treated S0 plants of RZ1, and which was identical with the untreated control (Figure 5a), indicating no immediate transpositional activity within the detecting resolution of gel-blotting for any of the tested TEs in the somatic leaf cells of the etoposide-treated S0 plants. Nevertheless, because the activity of many TEs is developmentally regulated [23,24], it was deemed possible that progenies of these S0 plants might show transposition of the TEs on condition the activity occurred in the germinal cells or progenitor cells thereof in the treated S0 plants. Indeed, we found that at least 10 of the 19 S1 plants showed rampant transpositional events for one of the eight studied TEs, i.e., *mPing*, as polymorphic patterns including both loss and gain of bands were detected in the gel-blotting probed by this element (Figure 5b, upper panel), consistent with the “cut-and-paste” model of transposition of this element [25]. In contrast, all the rest TEs showed only monomorphic blotting patterns (e.g., Figure 5b, lower panel for probe *Osr35*), denoting stability of these TEs even in progenies of the etoposide-treated plants. Similarly, at least 14 of the 15 S2 individuals derived from a single S1 plant (S1-7) showed further transpositions of *mPing* (Figure 5c, upper panel), although the particular S1 plant itself did not show *mPing* transposition. Taken together, our results indicated that subsequent to the etoposide treatment, activity of *mPing* was altered in such a way that they became transgenerationally mobilizing.

To further verify transposition of *mPing*, we performed *mPing*-specific transposon display (TD) analysis [19] for a larger number of etoposide-treated S0 (30 individuals), S1 (40 individuals) and S2 (30 individuals) plants of RZ1. Although no polymorphism in the *mPing-*specific TD profiles was observed in any of the 30 studied S0 plants, we detected a large number of loss and gain of bands in the S1 and S2 plants, denoting transpositional activity of *mPing*. We then isolated and sequenced a subset of these variant bands and used them as queries to blast against the reference rice genome of Nipponbare (http://rgp.dna.affrc.go.jp). We next designed locus-specific PCR primers (see Additional file 2) based on the matched Nipponbare sequence, which putatively encompassed the *mPing* copies representing either excision or reinsertion events in the S1 or S2 plants derived from the etoposide-treated S0 plants of RZ1. The results of PCR amplification using DNA of the untreated control plants of RZ1 as a template indicated that all five loci produced PCR amplicons of sizes consistent with harboring an internal *mPing* copy (430 bp in length), while all 34 loci that presumably represented reinsertion events in the S1 or S2 plants derived from the etoposide-treated S0 plants, and therefore should not contain a *mPing* copy in the untreated control, indeed had smaller-sized bands consistent with lacking of a *mPing* copy (data not shown).

**Figure 6:**
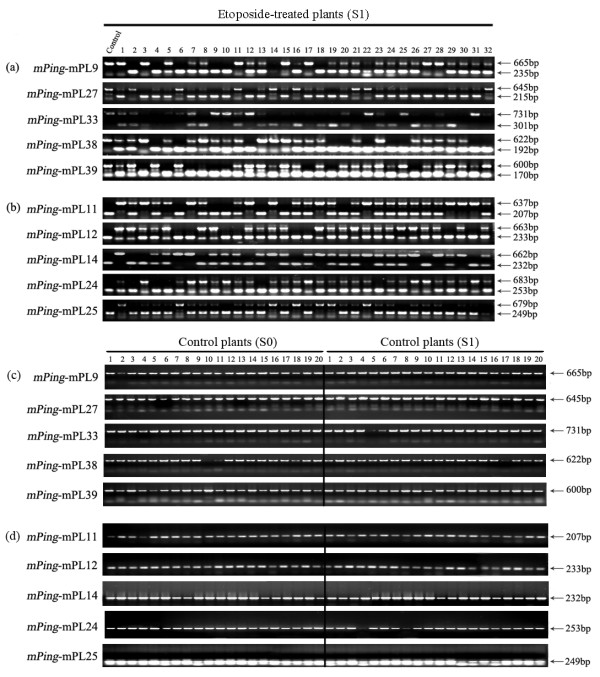
**Validation of excision and reinsertion events of *mPing *by locus-specific PCR amplification in 32 random S1 individuals derived from multiple etoposide-treated S0 plants of RZ1 (in which no evidence for either excision or reinsertion events of *mPing *was detected) and confirmation of *mPing *stability in two generations (S0 and S1) of the wild-type control plants of RZ1. (a)** The five *mPing*-containing loci in untreated control of RZ1 showing excisions in the various S1 individuals (depending on locus). Representatives of both the upper- and lower-bands were sequenced, which verified that length difference between the two bands for a given locus was exactly 430, i.e., the full length of *mPing,* as indicated on the right side of the figure. **(b)** Five of the 20 characterized *mPing*-empty loci in untreated control of RZ1 showing reinsertions in the various S1 individuals (depending on locus). Representatives of both the upper- and lower-bands were sequenced, which verified that the length difference between the two bands for a given locus was exactly 430, i.e., the full length of *mPing*, as indicated on the right side of the figure. **(c)** and **(d)** are amplification results by the same five *mPing*-containing loci and five *mPing*-devoiding loci as in **(a)** and **(b)**, respectively, on 40 individual plants of the wild-type control plants of RZ1 at two generations (S0 and S1). Labeling of the band sizes is the same as in **(a)** and **(b)**.

With these locus-specific, *mPing*-containing (n = 5) or -devoiding (n = 34) primer pairs, we first analyzed the etoposide-treated S0 plants (n = 30) of RZ1, and we found only monomorphic patterns identical with those of the control plants (data not shown), confirming that no transpositional events occurred immediately in the leaf somatic cells of the etoposide-treated S0 plants, consistent with the gel-blotting results. We next analyzed the 32 S1 plants (the same as used for TD analysis, described above) derived from the etoposide-treated S0 plants of RZ1, and found that all the five primer pairs harboring *mPing* showed evidence of excisions (Table 2) in portions of the S1 plants, as concomitant loss of the larger-sized band harboring a *mPing* and gain of a smaller-sized band lacking a *mPing* were detected in these plants, with different plants manifesting the changes at different loci (Figure 6a). Similarly, 20 of the 34 primer pairs (58.8%) detected *de novo* insertions in a large proportion of the 32 S1 plants relative to the RZ1 control plants (Table 3), as gain of a larger-sized band consistent with gaining a *mPing* and loss of a smaller-sized band originally devoiding of a *mPing* were detected in each of these cases (e.g., Figure 6b). Further analysis by the locus-specific PCR amplification on a set of 19 S2 plants derived from the studied S1 plants indicated that at least 14 additional new insertions were detected (Table 4), indicating mobility of the activated *mPing* was retained transgenerationally.

**Table 2:** **Characterization of five *mPing *excision sites isolated from the *mPing*-specific TD profiles in the progenies (S1 generation) of etoposide-treated RZ1 plants**

**Excision sites**	**Excision position ^a^**	**Locus-specific primers (5’-3’)**	**Excised from (S1 plant individuals)**	**Excision footprint**
mPT9	Chr.11; Position:	for: tactgccttttgctccatcc	2,4-10,12-14,16,18-26,29-32	gcaagtgaatacTTA **<** ***mPing*(430bp) >** TTAggactactttga
	22698635-22699300	rev: caggctttgccaatagaaca		gcaagtgaatacTAA------------ggactactttga
mPT27	Chr.1; Position:	for: tgtggttgtggtagctgcat	1,3-5,7-10,12-13,15,17-20,23-31	tgccatgtgctaTTA < ***mPing*(430bp)** > TTAtgcccggaggcc
	17840580-17841225	rev: ctgtaccgcacggcagtatt		tgccatgtgctaTTA------------tgcccggaggcc
mPL33	Chr.6; Position:	for: gaggcaggagattagggttg	1-3,7-8,11,13-21,23-24,26-30	tctaatggttcaTTA **<** ***mPing*(430bp) >** TTAgggggtagtggg
	13736451-13737182	rev: gacaatgcccactgttagga		tctaatggttcaTTA------------gggggtagtggg
mPL38	Chr.3; Position:	for: caacgcttcacctaaccaca	1-2,4-12,16-32	cagctacactctTTA **<** ***mPing*(430bp) >** TTAtgtggaagaaac
	23839760-23840381	rev: cggcacacagagaaatgatg		cagctacactctTTA------------tgtggaagaaac
mPL39	Chr.11; Position:	for: gtggtttcccatccgtcata	1,3,5,7-10,14,16-24,26-28,30-31	atatcagtacggTTA **<** ***mPing*(430bp) >** TTAagaaacccaaca
	11010652-11011251	rev: cggctttatcagtgcaaggt		atatcagtacggTTA------------agaaacccaaca

^a^ Based on BlastN at the NCBI website.

**Table 3:** **Characterization of 20 sites (isolated from the *mPing*-specific TD profiles) flanking de novo *mPing *reinsertions in the S1 progenies of etoposide-treated RZ1 plants**

**Insertion sites**	**Insertion position^a^**	**Locus-specific primers**	**Inserted into**	**TIRs (5’-3’)**	**TSDs (5’-3’)**
		**(5’-3’)**	**(S1 plant individuals)**		
*mPing*-mPL7	Chr.11; Position:	for: gaaactaacgcgtgcacaga	1,3-5,7-10,12,15-16,18-20,23,25-31	ggccagtcacaatgg	TAA
	25106489-25107127	rev: gcgattcagcataacaccaa			
*mPing*-mPL8	Chr.3; Position:	for: tcccattcaaagatgacgaa	1,3,6-12,14-22,25-31	ggccagtcacaatgg	TAA
	20981135-20981762	rev: gaacacgaaacaacagaacacc			
*mPing*-mPL11	Chr.8; Position:	for: atctccatcccctcacgac	1-5,7-8,11-12,14,16-19,21-32	ggccagtcacaatgg	TTA
	1013283-1013920	rev: aaaagtgtcggaagctctgc			
*mPing*-mPL12	Chr.7; Position:	for: gcacaggctccaagacgta	1-5,8-9,12-15,18-28,30,32	ggccagtcacaatgg	TAA
	4559135- 4559798	rev: aaaaactgaccgttggatgg			
*mPing*-mPL13	Chr.4; Position:	for: ggcaatggtgattcgttga	16-17,24-26,28-30	ggccagtcacaatgg	TAA
	34469107-34469738	rev: tgcatgagagccaatactcc			
*mPing*-mPL14	Chr.12; Position:	for: cccatttgaataccggatga	1,4-7,9-11,13-15,17-26,28-30,32	ggccagtcacaatgg	TAA
	2733377-2734039	rev: ctgggcaacttggagtacg			
*mPing*-mPL16	Chr.3; Position:	for: tttgcctttctgctgatcct	7,12-13,15,17-18,20-22,25-26,30,32	ggccagtcacaatgg	TAA
	34531398-34532060	rev: aacgatgccaaagtatgctg			
*mPing*-mPL17	Chr.3; Position:	for: gcaggcagatgttgatggta	1,3,6-9,11,14-15,18,20-23,27,30,32	ggccagtcacaatgg	TTA
	9406004-9406679	rev: tttgcatgcttgcttggtat			
*mPing*-mPL18	Chr.3; Position:	for: agcgatggtgcattggttat	1-33	ggccagtcacaatgg	TAA
	9647556-9548216	rev: ggaagctgctgcttttgaag			
*mPing*-mPL19	Chr.1; Position:	for: cgaatgcatcgataccactta	2-6,13,25-32	ggccagtcacaatgg	TTA
	25587854-25588476	rev: taatggcccaattcaatgct			
*mPing*-mPL20	Chr.12; Position:	for: tcaagaacagtgccaactcg	1,4-7,9-11,14-20,22-24,26,28-30,32	ggccagtcacaatgg	TTA
	3284650-3285306	rev: catacgccctattccgttgt			
*mPing*-mPL21	Chr.4; Position:	for: gtggagaaaatgggtgagga	1-3,7-8,17-22,27-30,32	ggccagtcacaatgg	TTA
	34083700-34084342	rev: tacgggtgttgacatgaagc			
*mPing*-mPL22	Chr.2; Position:	for: aaacccacggtttgcttttt	1-8,11,16-17,22-26,30-32	ggccagtcacaatgg	TTA
	22543199-22543812	rev: ggaagacagagccactgagc			
*mPing*-mPL23	Chr.5; Position:	for: atgcaaagatttggtgagca	1-33	ggccagtcacaatgg	TTA
	19245393-19246079	rev: cccacacctttgatttttcg			
*mPing*-mPL24	Chr.3; Position:	for: catgtgcgtggaaaacagag	1,3,6-12,14-22,24-32	ggccagtcacaatgg	TTA
	21314690-21315373	rev: ggtgcggaacatgtcatcta			
*mPing*-mPL25	Chr.8; Position:	for: tgaggcattgaggtgcacta	1,2-911-24,26-28	ggccagtcacaatgg	TTA
	13161847-13162504	rev: cgctatattaatgccggttcc			
*mPing*-mPL28	Chr.2; Position:	for: tatctgagcgtgagcgtgtc	2-7,9-10,12,15,17-26,28-30,32	ggccagtcacaatgg	TAA
	29238347-29238998	rev: ttatttggggacgacctttg			
*mPing*-mPL31	Chr.6; Position:	for: gtccgatggatcctactggt	1-33	ggccagtcacaatgg	TAA
	30098290-30099010	rev: attaagcatgcatgggtgtg			
*mPing*-mPL32	Chr.8; Position:	for: tcctcctactcctccacagc	1-8,12-14,16-32	ggccagtcacaatgg	TAA
	4706504-4707237	rev: cacaacaggcaacctcaact			
*mPing*-mPL34	Chr.12; Position:	for: aatcgcgaaaatgaactctg	1-33	ggccagtcacaatgg	TAA
	9948317-9949045	rev: ggcacagctcctaacaggta			

^a^ Based on BlastN at the NCBI website.

**Table 4:** **Characterization of 14 additional sites (isolated from the *mPing*-specific TD profiles) flanking de novo *mPing *insertions in the S2 progenies of etoposide-treated plants**

**Insertion site**	**Insertion position^a^**	**Locus-specific primers**	**Inserted into**	**TIRs**	**TSDs (5’-3’)**
		**(5’-3’)**	**(S2 plant individuals)**	**(5’-3’)**	
*mPing*- mPL1	Chr.4; Position:	for: tggtttgctgggacatgtaa	3-15,17,19	ggccagtcacaatgg	TAA
	35202559-35203218	rev: gctcttgcataagagccaaca			
*mPing*- mPL2	Chr.2; Position:	for: gcagccagtacgtagcacag	2,9,10	ggccagtcacaatgg	TAA
	28002407-28003049	rev: acgaacgtgggctgttttag			
*mPing*- mPL3	Chr.5; Position:	for: tttgtcggcgtctactccat	2,9,10	ggccagtcacaatgg	TAA
	22152251-22152950	rev: tttgcagctggcttatagca			
*mPing*- mPL4	Chr.3; Position:	for: gctcgtggctgaagacctta	2,9,10	ggccagtcacaatgg	TTA
	9219105-9219744	rev: tcgtctctcggtgacacagt			
*mPing*- mPL5	Chr.12; Position:	for: atgtgcactgtgcctggtag	2	ggccagtcacaatgg	TTA
	838497-839154	rev: tctcgctctttcagtgagca			
*mPing*- mPL6	Chr.8; Position:	for: cggagcacggagtacttatca	2,9,11-12,15-16	ggccagtcacaatgg	TAA
	28053221-28053889	rev: gctctaaatcacctagccaacg			
*mPing*- mPL10	Chr.1; Position:	for: tggctggtccttaccttttg	10-12,14-17,19	ggccagtcacaatgg	TTA
	23659253-23659876	rev: gacgtggagaggtggaagag			
*mPing*- mPL15	Chr.3; Position:	for: ttgagagcatccacaacgaa	2,9,10	ggccagtcacaatgg	TTA
	12714414-12715082	rev: atcggcattagcacaaagga			
*mPing*- mPL26	Chr.2; Position:	for: caaagccaaaacaaggatgc	9,10	ggccagtcacaatgg	TTA
	13161847-13162504	rev: aagggcgcatattagcaaaa			
*mPing*- mPL29	Chr.4; Position:	for: acaatcaatggcttccttgc	2,10	ggccagtcacaatgg	TTA
	32802687-32803380	rev: ccaagtgtcatgcctgctta			
*mPing*- mPL30	Chr.1; Position:	for: gtgggaagtgatgaggagga	2-4,8-10,18	ggccagtcacaatgg	TTA
	30258277-30258933	rev: cgcgggggattagaatactt			
*mPing*- mPL35	Chr.8; Position:	for: aaagagaaaagcagcggact	2	ggccagtcacaatgg	TAA
	28053307-28054025	rev: aaatgacggttttgttttgc			
*mPing*- mPL36	Chr.11; Position:	for: gccgcgagctaatgatagtt	2,8,10	ggccagtcacaatgg	TTA
	392454-393170	rev: gtaaccctgccctgactcat			
*mPing*- mPL37	Chr.3; Position:	for: tttacgtcaggggaatggac	1,7,8,10,14,16-17	ggccagtcacaatgg	TAA
	17530417-17531150	rev: tccgcgttcttcagtttcta			

^a^ Based on BlastN at the NCBI website.

To test the remote possibility that *mPing* at these loci in the specific genotype (RZ1) might be intrinsically unstable irrespective to the drug treatment, we further tested 40 individual plants (20 and 20 from the S0 and S1 generations, respectively) of the wild-type RZ1 with the same 10 loci described above. We detected only monomorphic patterns across all tested individual plants (Figure 6, c and d), indicating lack of any excision or insertion event at these loci in these plants, thus lending further support to the conclusion that mPing mobility was causally linked to etoposide treatment.

### S1 and S2 plants descended from the etoposide-treated S0 plants of RZ1 showed heritable phenotypic variations

**Figure 7:**
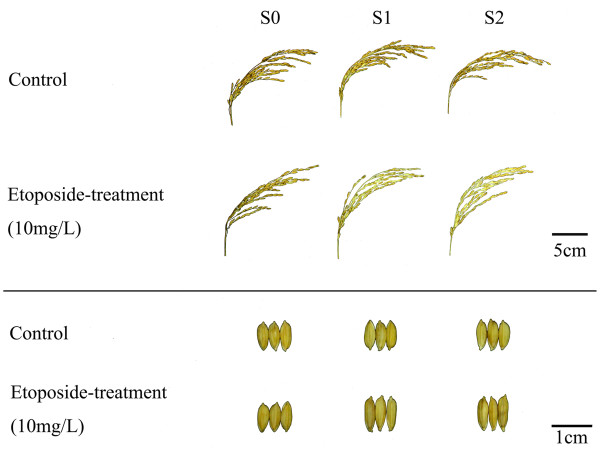
**Illustrations of heritable alteration in fertility (upper panel) and kernel-shape (lower-panel) in progenies (S1 and S2) of the etoposide-treated S0 plants of RZ1.** Reduced fertility was observed in the S1 and S2 progenies of the etoposide-treated S0 plants of RZ1, the kernel-shape of which also became elongated. Both altered traits were inheritable (at least from S1 to S2).

**Figure 8:**
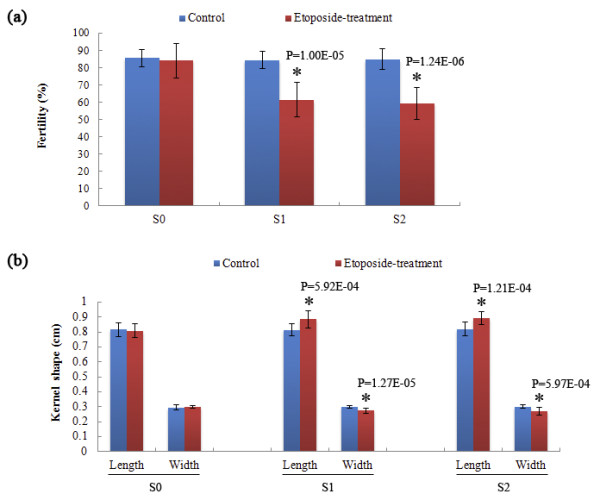
**Quantification of heritable alteration in fertility (a) and kernel-shape (b) in progenies (S1 and S2) of the etoposide-treated S0 plants of RZ1.** Thirty random samples (panicles for fertility and kernels for shape) were measured and quantified. Statistical test was conducted by the Independent-Sample T Test using SPSS 17.0 software (see Additional file 3).

We examined the phenotypic stability in the etoposide-treated S0 plants and their S1 and S2 progenies for all six genotypes. We found that only some of the S1 and S2 plants descended from the etoposide-treated S0 plants of RZ1 showed clear phenotypic variations in several traits, with reduced seed-setting (compromised fertility) and kernel shape (elongated kernel length but reduced width) being the most conspicuous (Figures 7 and 8; see Additional file 3). Moreover, the altered phenotypic traits were stably inherited from S1 to S2 (Figures 7 and 8), suggesting that they were likely to have a genetic and/or epigenetic basis rather than being caused by physiological perturbations by the drug treatments applied in the S0 generation.

## Discussion

As a topoisomerase II inhibitor, the major biological function of etoposide is to inhibit DNA synthesis, and thereby, resulting in accumulation of double-stranded DNA breaks (DSBs) [1,2]. Consequently, as documented by previous studies in animal and human cells, etoposide constitutes a genotoxic stress which may also induce genomic instability indirectly by causing transpositional activation of otherwise quiescent transposable elements (TEs), and hence generating insertional mutagenesis [3]. Most plant genomes harbor potentially active TEs which can be induced to become transpositionally active under specific conditions [4,5]. Nonetheless, no information is available regarding whether the chemical etoposide may produce similar effects in plant cells as it does in animals [3].

We have shown in this study that etoposide may indeed cause rampant transposition of a MITE transposon, *mPing*, endogenous to the rice genome [25]. However, the activation of *mPing* occurred in a strictly genotype-dependent manner, as only one of the six tested genotypes showed this phenomenon. In addition, the transpositional events did not occur in somatic cells of the immediately treated plants (S0); rather, excision and reinsertion events were detected only in selfed progenies (S1 and S2) of the etoposide-treated S0 plants. This is consistent with developmental regulation of most plant TEs [26]. Thus, for example, if the transpositions of *mPing* occurred at the gametogenesis stage of the etoposide-treated S0 plants, then the events can not be detected in leaf tissue of treated S0 plants but should be detectable in any sporophyte tissues of the S1 plants.

Authenticity of transposition rather than genomic rearrangements was verified by multiple independent assays including gel-blotting, *mPing*-specific TD, sequencing and locus-specific PCR amplification. The fact that only one of the eight analyzed TEs with transpositional potentiality was actually activated is in accord with the recent finding that the controlling mechanisms of TEs in plant genomes are highly individualized by diverse repressive epigenetic modifications [4,27]. The mobilization of only *mPing* without entailing concomitant transpositional activation of its TPase donors *Ping* or *Pong* is also congruent with previous findings on this element [21]. This is understandable as only transcriptional activation of its TPase donor should be sufficient for *mPing* transposition. Sequencing of the newly inserted *mPing* copies indicated that, on the one hand, the insertions were randomly distributed with regard to the 12 chromosomes of the rice genome, and on the other hand, the great majority of the insertion sites were landed within low-copy, genic regions. Both characteristics are in line with known propensity of newly induced *mPing* insertions by various inductive conditions [19,21,28].

A hallmark of epigenetic modifications lies in its metastability in response to internal or environmental perturbations. Importantly, alterations of at least some of the epigenetic modifications, e.g., cytosine DNA methylation, are known to produce transgenerational biological effects which could be relevant to coping with the particular stress condition [8-11]. This is a particularly pertinent issue in plants, as altered DNA methylation patterns are more readily transmissible to organismal generations through meiosis in plants than in animals, and thereby, the biological effects they dictate [14]. Therefore, it is apparently interesting to test whether the genotoxic effects of etoposide may also instigate epigenetic instabilities in addition to its genetic mutagenesis. Surprisingly, however, to our knowledge, no such information is available in any organism.

Thus, we have shown in this study for the first time in any organism that the topoisomerase II inhibitor etoposide is also epigenetically mutagenic in the sense that the chemical generated immediate changes in DNA methylation patterns in the leaf somatic cells of the treated S0 rice plants. However, this effect, at least in rice, is of variable penetrance with regard to genetic context, as only four out of the six studied genotypes showed evidence of methylation changes. Interestingly, nearly all the detected methylation changes by methylation-sensitive gel-blotting were found to occur only at the CHG sites, underscoring differential stability of CG vs. CHG methylation modifications in plants. It has been well-established that in plant genomes the three types of cytosine methylation patterns, CG, CHG and CHH, are maintained by distinct yet, to an extent, also overlapping DNA methyltransferases, with CG methylation being predominantly maintained by DNA Methyltransferase 1 (MET1), CHG methylation by Chromomethylase 3 (CMT3, a plant-specific DNA methyltransferase), and CHH methylation by Domains Rearranged Methyltransferase 2 (DRM2, a *de novo* DNA methyltransferases which is also partly responsible for *de novo* methylation of all cytosines) [29]. Therefore, it can be envisioned that the etoposide treatments may differentially affect activity or titration of these DNA methyltransferases and result in the observed predominant CHG methylation changes. Nonetheless, based on the higher resolution analytical approach, i.e., bisulfite sequencing, which was capable of revealing methylation change of any single cytosine within the analyzed region, we have found that, for both analyzed loci, all three types of cytosine methylation patterns, CG, CHG and CHH, underwent changes due to the etoposide treatment. But taking both loci together, it still holds that CHG methylation appeared more prone to changing as a result of the etoposide treatment than CG methylation, which is consistent with the situation of other environmental stress-induced cytosine methylation changes in plants [18].

The detected methylation changes included both decrease (hypo) and increase (hyper) in methylation, but with the former seemingly more predominant than the latter. This is consistent with the perturbation of otherwise fine-tuned activity of the various DNA methyltransferases that are responsible for faithfully maintaining the inheritable methylation patterns. However, it has been shown that loss of methylation of certain type (e.g., CG) may activate alternative back-up cellular systems, which in turn may catalyze hypermethylation of other types of methylation (e.g., CHH) and produce aberrant methylation patterns [30]. This scenario may explain the detected hypermethylation in the bisulfite sequencing results.

It seems surprising that little difference was detected between the two concentrations (10 mg/L vs. 20 mg/L) of the etoposide treatment with regard to the induced DNA methylation changes. It has been established in human cells that each molecule of etoposide stabilizes only one single-stranded DNA break [1]. Therefore, it can be deduced that if 10 mg/L has already reached the saturated concentration for the germinating rice seeds, then no difference should be expected with elevated concentrations.

Phenotypic examination under paddy-field condition of the etoposide-treated S0 rice plants and their selfed S1 and S2 generations showed that of the six genotypes, only S1 and S2 plants of RZ1 showed clear and heritable (at least between S1 and S2) variations in several phenotypic traits like fertility and kernel-shape. Because *mPing* transpositional activity was concomitantly detected in these plants, it suggests that the co-occurrence of the two phenomena may be more than coincidental. Additional investigations are needed to explore the correlative or causal relationships between the two phenomena.

## Conclusions

Results of this study have demonstrated that the eukaryotic topoisomerase II-blocking drug, etoposide, imposes a similar genotoxic stress on plant cells as it does on animal and human cells. In rice, rampant mobilization of an endogenous MITE transposon, *mPing*, was detected in selfed progenies of the etoposide-treated plants in one of the studied cultivars, pointing to transgenerational genomic instability by the drug treatment. In addition, epigenetic instability in the form of altered DNA methylation patterns was even more generally observed in somatic cells of rice plants shortly after the etoposide treatment, indicating immediate epigenetic effects of the drug. However, it should be noted that the mutagenic effects of etoposide at both the genetic and epigenetic levels are genotype- as well as organismal generation-dependent.

## Materials and Methods

### Plant materials

Seeds of six cultivated rice (*Oryza sativa* L.) genotypes, including the standard laboratory cultivars for each of the two subspecies, *japonica* (Nipponbare) and *indica* (93-11), were used. Four additional genotypes (Matsumae, RZ1, RZ2 and RZ35) all belonging to the *japonica* subspecies are akin to each other, as the three recombinant inbred lines (RILs), RZ1, RZ2 and RZ35, are derived from the same rice genotype (Matsumae) and a wild rice species, *Zizania Latifolia* Griseb [31]. All the genotypes were maintained in our hands by strict selfing.

### Etoposide treatment

Uniform seeds of each of the six rice genotypes were germinated in three different solutions, double-distilled water (used as the control), 10 mg/L etoposide solution and 20 mg/L etoposide solution, respectively. These two concentrations of etoposide were chosen on the basis of both previous studies in animal cells and our preliminary experiments in rice (unpublished data). The solutions were changed every day, for seven days. The germinating seeds were allowed to develop into seedlings in petri dishes and then be transplanted to regular paddy-fields. Randomly selected individuals (designated as S0) were tagged and later analyzed. Seeds were collected from each individual plant and designated as S1 for selfed progenies of both the controls and etoposide-treated plants. Similarly, we designated selfed progenies of the S1 generation as S2. We extracted genomic DNAs from leaves of all these plant materials for molecular analyses.

### Conventional and methylation-sensitive gel-blotting analyses

Conventional and methylation-sensitive gel-blotting analyses followed similar protocols, the only difference lied in using different restriction enzymes for DNA digestion. The former employed *Xba*I as restriction enzyme, while the latter employed a pair of methylation-sensitive isoschizomers, *Hpa*II and *Msp*I instead. All restriction enzymes were purchased from New England Biolabs Inc. (Beverly, Massachusetts). Specifically, genomic DNA was isolated from expanded leaves of individual plants by a modified CTAB method [32] and purified by phenol extraction. The DNA was digested by restriction enzyme(s). To ensure complete digestion, an excess of enzyme (10 units enzyme per μg DNA) was used and the incubation time was extended to 48 h. Digested DNA was run through 1% agarose gel and transferred onto Hybond N^+^ nylon membranes (Amersham Pharmacia Biotech, Piscataway, New Jersey) by the alkaline transfer method recommended by the supplier. For probes, we used eight transposable elements (TEs) for conventional gel-blotting, and eight transposable elements (TEs) and seven known-function, protein-encoding genes for methylation-sensitive gel-blotting. Specific primers (see Additional file 4) for amplifying each of these probes were designed based on sequences deposited at GenBank, and the fragments were amplified by PCR at an annealing temperature of 58-60°C. The probe fragments were verified by sequencing, and then gel-purified and labeled with fluorescein-11-dUTP by the Gene Images Random Prime-labeling Module (Amersham Pharmacia Biotech). Hybridization signals were detected by the Gene Images CDP-Star detection module (Amersham Pharmacia Biotech) after washing at a stringency of 0.2× SSC, 0.1% SDS for 2× 50 min. Finally, the filters were exposed to X-ray films.

### Bisulfite sequencing analysis

Bisulfite sequencing was carried out as described [33], which involved bisulfite treatment of single stranded DNA by the EZ DNA Methylation-Gold^TM^ Kit (ZYMO Research Corporation). Bisulfite treatment converts unmethylated cytosines into uracils while methylated cytosines remain unconverted. After treatment, the region of interest was PCR amplified by the designed primers for each of the two studied TEs, *Tos17* and *Osr36* (see Additional file 5), and the PCR products were cloned and sequenced. The PCR amplification of the converted cytosine (to uracil) would result in the replacement of uracil with thymine. Analyses of the bisulfite-sequencing results were conducted at the Kismeth website (http://katahdin.mssm.edu/kismeth).

### Transposon display

Transposon display (TD) [34] was performed by combining the *mPing* sub-terminal-specific primers [25] with *Mse*I-adaptor-specific primers (see Additional file 6) and the amplicons were visualized on 5% polyacrylamide gels after silver-staining. Compared with the control plants, novel bands and lost bands in TD gels of etoposide treated plants (S0, S1 and S2) were considered as putative *mPing* de novo insertions and excisions, respectively. A subset of the variable bands were eluted, cloned and sequenced. The sequences were then blasted against the whole genome sequence of rice cv. Nipponbare (http://rgp.dna.affrc.go.jp). Based on this analysis, a set of locus-specific primers were designed, as detailed below.

#### PCR-based locus assay on *mPing* transposition

To validate the putative transpositions of *mPing* in the S0, S1 and S2 generations of the etoposide-treated rice plants, revealed in the TD analysis, a set of 39 pairs of locus-specific primers each containing an intact *mPing* (see Additional file 2) were used for PCR amplification at annealing temperatures ranging from 56°C to 60°C depending on the primers. The amplicons were visualized by ethidium bromide staining after electrophoresis through 1% agarose gels. The identified excision and insertion sites of *mPing* were isolated, cloned and sequenced for confirmation.

## Authors' contributions

XY and YY carried out major parts of the experiments, analyzed the data and drafted the manuscript. XL, LJ, CZ and XO participated in all the experiments. KO edited the manuscript. BL designed the work and finalized the manuscript. All authors read and approved the final manuscript.

## Supplementary Material

Additional file 1 The nucleotide sequence of the bisulfite sequenced region for each of the two TEs. Click here for file

Additional file 2 Sequences of the 39 pairs of primers for mPing locusspecific PCR amplification. Click here for file

Additional file 3 Statistical analysis of kernel shape and fertility between etoposide-treated plants and their controls in each generation. Click here for file

Additional file 4 The studied probe sequences and primers for probe fragment amplification in gel-blotting analysis. Click here for file

Additional file 5 Primers for bisulfite sequencing. Click here for file

Additional file 6 Adapters, pre-/selective amplification primers for transposon display (TD). Click here for file
