# Supplementary material for: Changes in DNA methylation and transgenerational mobilization of a transposable element (mPing) by the Topoisomerase II inhibitor, Etoposide, in rice
Source: BMC Plant Biol. 2012 Apr 9;12:48. doi: 10.1186/1471-2229-12-48 (PMC3480845; doi:10.1186/1471-2229-12-48)
Supplement: Additional file 1 — The nucleotide sequence of the bisulfite sequenced region for each of the two TEs. [file 1471-2229-12-48-S1.doc]

**Additional file 1** The nucleotide sequence of the bisulfite sequenced region for each of the two TEs*

| **Sequence name** | **Sequence** |
| --- | --- |
| *Tos17* | (**162**)TATATACAAGCTAATGTACTGTATAGTTGGCCCATGTCCAGCCCATCGGATGTCCAGTCCATTGGATCTTGTATCTTGTATATACTTCTCTATTGCTAATACTATTGTTAGGTTGCAAGTTAGTTAAGA(291)TGGTATCAGAGCAATGGTCTGAACCCTAGCTGCCAATTCCCCATCGCCGGCCGGCGCGGCGGCGTGCCGCCGGCGTTTCACTGCTAAGCAACCTCCTCCAATTCATATTTATCCACTGCTCCACTG(**417**) |
| *Osr36* | (**59688**)TGGTCATGTAACACTACGCCACCGCGAAACTCGCCGCCGACGAGAGAGAGAGATATTTAGACGAGATCGGAGTTGGGGACGAAGAGAAACAGAGCTGAGGCAGAGGAGAGACAGAGTTTGGAGAGAGATAGATTCGATTTCTTTTGCCAGGTTGGCCACAGCCATTCTCTCTCTCGAGTATTTATCTCCTGATTACATGGGCCGTTGGCCCAAACGATTTAAGGCACGCAGGCCGCATTCTATTTGATAAAGTCTAGCCCAAAACACACACACTATCATTCACAGTTAAACCTCTTTCTCAGTGTCACCATTGCCGGTCATGACAGTGCCGCCCGCAGGAGTTTCAGCTTGTCCCCAAGCTGGAGCATCTG(**60114**) |

Note: For *Tos17,* the analyzed region is 255 bp, with the first 129 bp being a portion of the 5'-LTR whereas the rest 126 bp being a portion of body-region (underlined); for *Osr36*, the whole region (426 bp) is within the 5'-LTR region of *Osr36.*
