# Supplementary material for: Changes in DNA methylation and transgenerational mobilization of a transposable element (mPing) by the Topoisomerase II inhibitor, Etoposide, in rice
Source: BMC Plant Biol. 2012 Apr 9;12:48. doi: 10.1186/1471-2229-12-48 (PMC3480845; doi:10.1186/1471-2229-12-48)
Supplement: Additional file 2 — Sequences of the 39 pairs of primers for mPing locusspecific PCR amplification. [file 1471-2229-12-48-S2.doc]

**Additional file 2** Sequence of the 39 pairs of primers for *mPing* locus-specific PCR amplification

| **Primer** | **Forward primer** | **Reverse primer** |
| --- | --- | --- |
| *mPing*-mPL1 | 5’-tggtttgctgggacatgtaa | 5’-gctcttgcataagagccaaca |
| *mPing*-mPL2 | 5’-gcagccagtacgtagcacag | 5’-acgaacgtgggctgttttag |
| *mPing*-mPL3 | 5’-tttgtcggcgtctactccat | 5’-tttgcagctggcttatagca |
| *mPing*-mPL4 | 5’-gctcgtggctgaagacctta | 5’-tcgtctctcggtgacacagt |
| *mPing*-mPL5 | 5’-atgtgcactgtgcctggtag | 5’-tctcgctctttcagtgagca |
| *mPing*-mPL6 | 5’-cggagcacggagtacttatca | 5’-gctctaaatcacctagccaacg |
| *mPing*-mPL7 | 5’-gaaactaacgcgtgcacaga | 5’-gcgattcagcataacaccaa |
| *mPing*-mPL8 | 5’-tcccattcaaagatgacgaa | 5’-gaacacgaaacaacagaacacc |
| *mPing*-mPL9 | 5’-tactgccttttgctccatcc | 5’-caggctttgccaatagaaca |
| *mPing*-mPL10 | 5’-tggctggtccttaccttttg | 5’-gacgtggagaggtggaagag |
| *mPing*-mPL11 | 5’-atctccatcccctcacgac | 5’-aaaagtgtcggaagctctgc |
| *mPing*-mPL12 | 5’-gcacaggctccaagacgta | 5’-aaaaactgaccgttggatgg |
| *mPing*-mPL13 | 5’-ggcaatggtgattcgttga | 5’-tgcatgagagccaatactcc |
| *mPing*-mPL14 | 5’-cccatttgaataccggatga | 5’-ctgggcaacttggagtacg |
| *mPing*-mPL15 | 5’-ttgagagcatccacaacgaa | 5’-atcggcattagcacaaagga |
| *mPing*-mPL16 | 5’-tttgcctttctgctgatcct | 5’-aacgatgccaaagtatgctg |
| *mPing*-mPL17 | 5’-gcaggcagatgttgatggta | 5’-tttgcatgcttgcttggtat |
| *mPing*-mPL18 | 5’-agcgatggtgcattggttat | 5’-ggaagctgctgcttttgaag |
| *mPing*-mPL19 | 5’-cgaatgcatcgataccactta | 5’-taatggcccaattcaatgct |
| *mPing*-mPL20 | 5’-tcaagaacagtgccaactcg | 5’-catacgccctattccgttgt |
| *mPing*-mPL21 | 5’-gtggagaaaatgggtgagga | 5’-tacgggtgttgacatgaagc |
| *mPing*-mPL22 | 5’-aaacccacggtttgcttttt | 5’-ggaagacagagccactgagc |
| *mPing*-mPL23 | 5’-atgcaaagatttggtgagca | 5’-cccacacctttgatttttcg |
| *mPing*-mPL24 | 5’-catgtgcgtggaaaacagag | 5’-ggtgcggaacatgtcatcta |
| *mPing*-mPL25 | 5’-tgaggcattgaggtgcacta | 5’-cgctatattaatgccggttcc |
| *mPing*-mPL26 | 5’-caaagccaaaacaaggatgc | 5’-aagggcgcatattagcaaaa |
| *mPing*-mPL27 | 5’-tgtggttgtggtagctgcat | 5’-ctgtaccgcacggcagtatt |
| *mPing*-mPL28 | 5’-tatctgagcgtgagcgtgtc | 5’-ttatttggggacgacctttg |
| *mPing*-mPL29 | 5’-acaatcaatggcttccttgc | 5’-ccaagtgtcatgcctgctta |
| *mPing*-mPL30 | 5’-gtgggaagtgatgaggagga | 5’-cgcgggggattagaatactt |
| *mPing*-mPL31 | 5’-gtccgatggatcctactggt | 5’-attaagcatgcatgggtgtg |
| *mPing*-mPL32 | 5’-tcctcctactcctccacagc | 5’-cacaacaggcaacctcaact |
| *mPing*-mPL33 | 5’-gaggcaggagattagggttg | 5’-gacaatgcccactgttagga |
| *mPing*-mPL34 | 5’-aatcgcgaaaatgaactctg | 5’-ggcacagctcctaacaggta |
| *mPing*-mPL35 | 5’-aaagagaaaagcagcggact | 5’-aaatgacggttttgttttgc |
| *mPing*-mPL36 | 5’-gccgcgagctaatgatagtt | 5’-gtaaccctgccctgactcat |
| *mPing*-mPL37 | 5’-tttacgtcaggggaatggac | 5’-tccgcgttcttcagtttcta |
| *mPing*-mPL38 | 5’-caacgcttcacctaaccaca | 5’-cggcacacagagaaatgatg |
| *mPing*-mPL39 | 5’-gtggtttcccatccgtcata | 5’-cggctttatcagtgcaaggt |
