# Supplementary material for: Changes in DNA methylation and transgenerational mobilization of a transposable element (mPing) by the Topoisomerase II inhibitor, Etoposide, in rice
Source: BMC Plant Biol. 2012 Apr 9;12:48. doi: 10.1186/1471-2229-12-48 (PMC3480845; doi:10.1186/1471-2229-12-48)
Supplement: Additional file 3 — Statistical analysis of kernel shape and fertility between etoposide-treated plants and their controls in each generation. [file 1471-2229-12-48-S3.doc]

**Additional file 3**

Statistical analysis of kernel shape and fertility between etoposide-treated plants and their controls in each generation were performed by Independent-Sample T Test using SPSS 17.0 software.

t=

| Kernel shape | S0 | S1 | S2 |
| --- | --- | --- | --- |
| Length | 0.648 | 5.92E-04* | 1.21E-04* |
| Width | 0.379 | 1.27E-05* | 5.97E-04* |

* The mean difference is significant at the 0.01 level.

|  | S0 | S1 | S2 |
| --- | --- | --- | --- |
| Fertility | 0.700 | 1.00E-05* | 1.24E-06* |

* The mean difference is significant at the 0.01 level.

Note: for kernels: *n*_1_=30 (number of kernels from control plants); *n*_2_=30 (number of kernels from etoposide treated-plants); for fertility: *n*_1_=30 (number of panicles of control plants); *n*_2_=30 (number of panicles of episode treated-plants)
