# Supplementary material for: Changes in DNA methylation and transgenerational mobilization of a transposable element (mPing) by the Topoisomerase II inhibitor, Etoposide, in rice
Source: BMC Plant Biol. 2012 Apr 9;12:48. doi: 10.1186/1471-2229-12-48 (PMC3480845; doi:10.1186/1471-2229-12-48)
Supplement: Additional file 4 — The studied probe sequences and primers for probe fragment amplification in gel-blotting analysis. [file 1471-2229-12-48-S4.doc]

**Additional file 4** The studied probe sequences, and primers for probe fragment amplification in DNA gel-blotting analysis

| **Probe** | **Genbank No. accession** | **Forward primer (5’-3’)** | **Reverse primer (5’-3’)** |
| --- | --- | --- | --- |
| *Tos17* | AC087545 | aaagggaaactcagcgaaca | gagggcacatagtggagagc |
| *Osr2* | AL442110 | cacaccagcaccaagtccta | tcgatcgctttaggttgctt |
| *Osr23* | AP002843 | gccggtcttgatgatgagtt | ttgaacagacgctccacaag |
| *Osr35* | AC068924 | tgatgtggtccttgagtcca | attctcttggcttggctgtg |
| *Osr36* | AP001551 | ccctgaatccaccaagaaaa | ggcagtctcgagaaggtgac |
| *Osr42* | AF458768 | ccacagatcatcatttctgacc | ccccttgaagactgacttgc |
| *Ping-specific* | AB087616 | ctacggagtacaccgcaacc | aatggattgcctactgctgact |
| *Pong-specific* | BK000586 | ggggtgaaacagcattgaga | tgtggttgcaaagaagacca |
| *mPing* | AP005628 | gtcacaatgggggtttcact | ggccagtcacaatggctagt |
| *Homeobox gene* | AB007627 | ttgatggaaatgatgggtca | actgcatcgtgcatcaaaac |
| *Binding* | X88798 | agaatgccactcctcctgtg | gtcctcccttctgtgctgag |
| *CAL-2* | AK069341 | aaactgtccccttccgagtt | cgcttcctcctcgtgtaaag |
| *CAL-11* | X81393 | atggactcccaatcaactcg | ctccatgaccccagtgagat |
| *Elongation factor* | D12821 | acctctccggcaagacctac | ttacaagccgctctgcagtt |
| *OsCDPK protein protein* | AY144497 | ccagctggcaactatcacaa | agatcatgcaccacctctcc |
| *CDPK-related protein kinase* | AP004380 | cagacatggggtgaggtttt | ccaccatagagccctgacat |
