# Supplementary material for: Changes in DNA methylation and transgenerational mobilization of a transposable element (mPing) by the Topoisomerase II inhibitor, Etoposide, in rice
Source: BMC Plant Biol. 2012 Apr 9;12:48. doi: 10.1186/1471-2229-12-48 (PMC3480845; doi:10.1186/1471-2229-12-48)
Supplement: Additional file 5 — Primers for bisulfite sequencing. [file 1471-2229-12-48-S5.doc]

**Additional file 5 Primers for bisulfite sequencing**

| **Locus** | **Chr.** | **Primer sequence** | **Size of product (bp)** |
| --- | --- | --- | --- |
| *Tos17* | 7 | forward: 5’-GATTGTYAGTGTGTGTTGTTAAATA  reverse: 5’-TARCCCACRARRCRACRRTRAAAARRACA | 307 |
| *Osr36* | 1 | forward: 5’-YTGAAAAGAATTAGTGAYTAGTTAGGTG  reverse: 5’-ACTATCATRTTRTRAARACTCRCTTTCCA | 426 |
