# Supplementary material for: Changes in DNA methylation and transgenerational mobilization of a transposable element (mPing) by the Topoisomerase II inhibitor, Etoposide, in rice
Source: BMC Plant Biol. 2012 Apr 9;12:48. doi: 10.1186/1471-2229-12-48 (PMC3480845; doi:10.1186/1471-2229-12-48)
Supplement: Additional file 6 — Adapters, pre-/selective amplification primers for transposon display (TD). [file 1471-2229-12-48-S6.doc]

**Additional file 6** Adapters, pre-/selective amplification primers for transposon display (TD)

| **Primer** | **Sequence** |
| --- | --- |
| *mPing1* | 5’-GCTGACGAGTTTCACCAGGATG |
| *mPing2* | 5’-TGTGCATGACACACCAGTG |
| *mPing3* | 5’-CAGTGAAACCCCCATTGTGAC |
| *MseI Adapter* | 5'- GACGATGAGTCCTGAG |
| *MseI＋0 pre-amplification primer* | 5'- GATGAGTCCTGAGTAA |
| *MseI＋3 selective amplification primers* | |
| *M-CAA* | 5’-GATGAGTCCTGAGTAACAA |
| *M-CAC* | 5’-GATGAGTCCTGAGTAACAC |
| *M-CAG* | 5’-GATGAGTCCTGAGTAACAG |
| *M-CAT* | 5’-GATGAGTCCTGAGTAACAT |
| *M-CTA* | 5’-GATGAGTCCTGAGTAACTA |
| *M-CTC* | 5’-GATGAGTCCTGAGTAACTC |
| *M-CTG* | 5’-GATGAGTCCTGAGTAACTG |
| *M-CTT* | 5’-GATGAGTCCTGAGTAACTT |
